# Supplementary material for: vmrseq: probabilistic modeling of single-cell methylation heterogeneity
Source: Genome Biol. 2024 Dec 30;25:321. doi: 10.1186/s13059-024-03457-7 (PMC11687023; doi:10.1186/s13059-024-03457-7)
Supplement: Supplementary file 3 — Additional file 3: Review history [file 13059_2024_3457_MOESM3_ESM.docx]

Review History

**First round of review**

**Reviewer 1**

**Were you able to assess all statistics in the manuscript, including the appropriateness of statistical tests used?**

**Yes:** No additional statistical review required.

**Were you able to directly test the methods?**

No.

**Comments to author:**

The authors presented an HMM-based method, called vmrseq, for detecting variable methylated regions (VMRs) in single-cell DNA methylation data. Their methods consist of two steps, involving kernel smoothing and two HMMs for the prediction of VMRs. The authors show superior performance on simulated data (mainly by Recall) and real data using a clustering metric. Furthermore, the authors provided one study detecting new potential insights into methylation-expression regulation. However, there are some problems in the evaluation, which should be accessed:

Major:
The authors wrote their method for simulating data, which is largely based on the same assumptions of their model. Thus, this evaluation potentially favors their model. For a fair comparison, the authors should also evaluate the simulation data that come with at least one other tool, e.g. scMET provides simulated data.

While the authors provide encouraging results using the nearest neighbor count score (as in scbs), other more classical evaluation metrics should be provided as well. Here, for example, the adjusted Rand index (as in scMET) or Silhouette score (without any clustering algorithm) could be used.

While vmrseq provided superior performance in the nearest neighbor count score, what happens if no VMR selection is performed and a simple binning approach, such as in SINDBAD for example is used as input for dimensionality reduction, e.g. by PCA?

The selection of parameters, such as α = 0.05, minimum CpG counts = 5, …, is not described very well. The authors claim that the selection works well in practice, but no quantitative results are shown. A small simulation case showing selected evaluation metrics for a couple of different choices of parameters could strengthen that claim.

vmrseq (stage 1 & 2) and the vmrseq CR (stage 1 only) algorithm provide very similar results in the simulation benchmarks (except for precision). This seems to be also true for real data (nearest neighbor score) and not only in the case of simulated chromosomes. However, the time complexity of optimizing a HMM and running it for each CR two times is probably much higher than stage 1 of the algorithm. Could stage 1 then not be enough for normal use-cases ?

Minor:
The variable C is introduced only in the time-complexity analysis but used already in equation (1)
Figure 1d is mentioned before Figure 1c
Several different variance cutoffs are used for multiple methods, but it is not explained why different cutoffs were used. Is that due to the difference in computing the variance?
very different variance cutoffs
The following section is discussed in the method section, but as it is an important limitation and information for a user, this should be rather part of the discussion and not the methods:
Only autosomes were included in this study since sex chromosomes have distinct methylation patterns (e.g., X-chromosome inactivation) where vmrseq may not be applicable. Follow-up work is needed to examine whether model parameters for sex chromosomes shall differ from autosomes.
Supplementary Figure 7-10 are very hard to read, due to the small font size.

Comment:
The Repository vmrseq-experiments contains user-paths and deprecated code which probably should not be there.

**Reviewer 2**

**Were you able to assess all statistics in the manuscript, including the appropriateness of statistical tests used?**

**Yes:** The statistical model was appropriate.

**Were you able to directly test the methods?**

Yes.

**Comments to author:**

vmrseq is a novel tool that identifies candidate regions with variable methylation patterns across cells. The tool is an advancement over previous tools which require a predefined set of regions. Specifically, vmrseq uses an HMM to decode Unmethylated (U) and Methylated (M) regions. The authors compare vmrseq against other tools in both simulated and real datasets and show improved performance. The software was able to be ran on a toy dataset. Overall, the manuscript is well written, compelling, and will be of interest to the broader single cell community. I only had a few relatively minor questions.

1) For the statement, "As a result, we remove the sites with an intermediate methylation level for every cell as a filtering step", does this mean that each item that is not 0 or 1 is considered missing?

2) Although no formal p-value was derived for comparing the one-group vs. two group models, would it be possible to generate an empirical distribution via permutation of the CpG sites?

3) Related to the results of Figure 3c,d, how were specific regions considered markers for a cell population? In standard single cell RNA-seq analysis, variable genes are chosen for clustering. However, a separate statistical test and metrices (e.g. Wilcoxon test in combination with a fold change cutoff) is used to find specific genes that are associated with each cluster. Can a similar paradigm be applied in the methylation clustering workflow?

4) Related to Figure 4b, it was stated that "the VMRs seemed to contain some variation associated with cell cycle, where the cells in G2M phase displayed a more pronounced clustering tendency than the other two phases". While it does appear that there is some correlation with cell cycle, it does not seem like the primary driving factor as cells from different parts are the cycle are represented in each part of the UMAP. If clustering is performed on the RNA space, how does the methylation clusters correlate with the RNA clusters?

5) The tool would be easier to install if it were deposited in a public R package repository such as CRAN or Bioconductor.

6) The online vignette is runnable using the supplied toy example. It would be nice to provide a vignette using a "real-world" dataset such as the ones analyzed in the paper which includes the downstream clustering analysis and annotation.

**Reviewer 3**

**Were you able to assess all statistics in the manuscript, including the appropriateness of statistical tests used?**

**Yes:** My review focused on the methodological aspects of the paper.

**Were you able to directly test the methods?**

Yes.

**Comments to author:**

The authors introduce vmrseq, a probabilistic approach to detect variable methylated regions (VMRs) associated cell-to-cell epigenetic heterogeneity. Unlike existing approaches, vmrseq does not rely on the use of a priori annotated regions (such as gene promoter) or sliding windows strategies. Instead, the authors implement a 2-stage approach in which an "scanning" step is first performed in order to identify candidate regions (CRs) in an unsupervised manner. In a second step, a hidden Markov model is used to flag VMRs among the pre-selected CRs (great to see that the authors discuss how the use of a 2-step approach prevents straightforward interpretation of p-values and error rates control). The performance of vmrseq was demonstrated using synthetic and real data, focusing on how the detection of VMRs can improve downstream clustering analyses.

The set of tools tailored to the properties of single cell methylation data vmrseq is somewhat limited. As such, vmrseq is a potentially important contribution in this area that complements existing approaches. However, as discussed below, some aspects of the methodology are unclear and the comparison with other approaches has some problematic aspects.

\#### Major comments

1. Data pre-processing.

a) Page 3 (lines 28-29) indicates that "sites with intermediate methylation level between 0 and 1 are filtered out for each cell". Does this mean that a different set of CpGs is considered for each cell? If so, how does this affect the construction of candidate regions? If the candidate regions are not consistent across cells, how does this affect the identification of VMRs?
b) Page 14 (lines 2-3) mentions that missing values are allowed in the input matrix. How are missing values treated when constructing the candidate regions and performing VMR selection?
c) The Methods states that the input for vmrseq is "processed and filtered whole-genome BSseq data". Beyond the filtering steps mentioned above, it would be helpful for potential users to have practical guidelines about more general quality control (e.g. to remove cells with poor data quality) that need to be applied prior to the use of vmrseq.

2. Synthetic data benchmark. The broad conclusion was that the sliding window was the most critical hyper-parameter choice. In particular, approaches using Smallwood and scMET (larger window size) performed worse than scbs and vmrseq (small window size). This comparison is problematic as the performance of the methods is confounded by the choice of window/step size. If you were to use smaller regions as input for Smallwood and scMET (e.g. 2000-bp as in scbs), do they perform better? Similarly, if you use a larger window size for scbs, does it perform worse? This issue is accentuated by the simulation setting, where a large number of the simulated VMRs were shorted than the window size used by some approaches (Fig S2). To make the comparison more fair, I suggest to match the window/step size when comparing different approaches. You could e.g. present different results for a small, medium and a large sliding window size for all methods.

3. Analysis of the mouse frontal cortex dataset.

a) The issues highlighted in the comment above also apply here. Again, the performance of the approaches shown in Figure 3b correlates with the choice of sliding window size. A fairer comparison will account for this by comparing performance of each method after matching window size.
b) Note that Kapourani et al analysed the same data using scMET and found better performance when using a more targeted approach in which input features were defined by a set of pre-annotated regions (e.g. gene promoters rather than a window size approach). It may be worth discussing such results and how a targeted approach (e.g. looking for VMRs only within gene promoters) could potentially improve performance across all methods (e.g. in Kapourani et al, methylation patters within distal H3K27ac and H3K4me1 peaks better characterised the sub-population structure in these data when comparing against gene promoters).
c) Similarly, the screening step used by vmrseq to identify CRs could be used as a pre-filtering step for other approaches too avoiding the use of sliding window approaches (of course, as the authors note, this would affect the interpretation of p-values, etc). It would be helpful to understand what step is more important: the initial screening step to identify CRs or the 2nd step (i.e. the HMM in vmrseq)?

4. Use of an annotated dataset to infer vmrseq parameters:

a) As noted in the discussion, the need of pre-annotated data can be problematic. As the same dataset was used for hyper-parameter tuning and benchmark, could this also affect the comparison shown in Figure 3b? Perhaps you could perform a sensitivity analysis to see how the results change if a different dataset the Luo et al data is excluded for hyper-parameter tuning (i.e. you only use the Liu et al data)?
b) Currently, all the information regarding the annotated dataset is provided in the supplement. It would be helpful to have a brief description in the main text.
c) When training the relevant models, who were the Luo et al and Lio et al datasets combined? Simple merge of all cells from both datasets?

5. Some aspects of the method are unclear and a substantial part of the methodological details are only provided in the supplement. This makes the paper harder to read.

a) Identification of candidate regions: when defining "5 adjacent CpGs", is there a minimum distance between the CpGs to be considered within the same CR?
b) Estimation of parameters for the "Empirical probability" model. It would be helpful to explicitly mention what parameters are estimated using the annotated data and a brief description of how these were obtained: maximum likelihood?
c) Page 16. Whilst it is easy to understand what do the hidden states represent in the one-grouping model specification, this is less clear for the two-grouping model specification. Please clarify the definition of bi-variate hidden states: what does each component of the vector $s_k$ represent?
d) Page 18. VMRs are defined for regions with a minimum 5 sites exhibiting the hidden state (0,1). The authors state that the number of sites (5 in their case) can be customised depending on the biological question. Please provide some guidance (examples?) of how this would be done.
e) Equation (5) is the ZIBB model trained in the same way as for the one-grouping model?

6. It would be helpful for users to understand the computational requirements. How do the different approaches compare in terms of scalability?

\#### Minor comments

1. Improvements for some Figures:
a) Figure 1b: define colour-scale for yellow lollipop. Missing?
b) Caption of Fig 1d. Unclear how the "homogenous cell population" was defined here. Is this synthetic data? Real data subsetted in some way?
c) Figure S7 is too small and difficult to read without zooming in.

2. I was able to install the R library from GitHub with no problem. I was also able to run the vignette. However, I got a couple of warnings after running `results <- vmrseq.fit(gr)`. Not sure this affects the performance of vmrseq, but worth checking why do they appear.

```
Warning messages:
1: In split.default(which(direction > 0), segments[direction > 0]) :
data length is not a multiple of split variable.

**Authors Response**

**Point-by-point responses to the reviewers’ comments:**

# Reviewer #1

Reviewer #1: The authors presented an HMM-based method, called vmrseq, for detecting variable methylated regions (VMRs) in single-cell DNA methylation data. Their methods consist of two steps, involving kernel smoothing and two HMMs for the prediction of VMRs. The authors show superior performance on simulated data (mainly by Recall) and real data using a clustering metric. Furthermore, the authors provided one study detecting new potential insights into methylation-expression regulation. However, there are some problems in the evaluation, which should be accessed:

*Reply: We thank the reviewer for providing supportive and constructive comments. We address all the questions and comments in our point-by-point response below.*

**Major:**

1. The authors wrote their method for simulating data, which is largely based on the same assumptions of their model. Thus, this evaluation potentially favors their model. For a fair comparison, the authors should also evaluate the simulation data that come with at least one other tool, e.g. scMET provides simulated data.

*Reply: Thank you for your comment. We had similar concerns prior to conducting the simulation studies. Thus, in the first simulation study presented in the main text, we utilized real-world experimental data and introduced synthetic VMRs generated using assumptions of the hidden Markov model (HMM) in vmrseq. Although the spiked-in VMRs relied on HMM assumptions, such as the spatial dependence structure and the model variance structure, the vast majority of CpGs in the chromosome used in this simulation were derived from actual experimental data and not simulated from the model.*

*We adopted the HMM model in vmrseq due to the absence of other tools that meet our specific requirements. While scMET, a Bayesian hierarchical model, does provide simulated data based on its assumptions, it is not capable of generating* ***site-level*** *data required for our analysis. Specifically, scMET can only provide the total count of methylated CpGs within predefined windows, which does not meet our need for detailed site-level data. Further, to the best of our knowledge, there are no existing models or functions in the literature that provide site-level single-cell bisulfite sequencing data.*

*With that said, we fully acknowledge that the simulation is in favor of vmrseq and we thank the reviewer for highlighting this limitation. In response, we included the following paragraph in the Discussion section of the revised manuscript to address this concern:*

*“We acknowledge that the evaluation through simulation studies potentially favors vmrseq, due to the fact that synthetic data were partly or fully generated according to assumptions of our model. Ideally, we would leverage alternative data-generative methods for a more unbiased method comparison and assessment. However, to the best of our knowledge, there are no existing models or functions in the literature that provide site-level single-cell bisulfite sequencing data. We recommend further evaluations using alternative methods if and when they become available.”*

2. While the authors provide encouraging results using the nearest neighbor count score (as in scbs), other more classical evaluation metrics should be provided as well. Here, for example, the adjusted Rand index (as in scMET) or Silhouette score (without any clustering algorithm) could be used.

*Reply: Thank you for the suggestion. In response, we have incorporated the Silhouette score* [*[1]*](https://www.zotero.org/google-docs/?WevhDF) *as an additional evaluation metric in our manuscript, supporting the encouraging results we reported using the nearest neighbor count score. We have included a new section in the supplementary materials (Additional File 1: Section S2.1) discussing the results obtained from the Silhouette score analysis as well as a corresponding sentence in the main text that links to the supplementary materials. The added sentence is as follows:*

*“Similar conclusions were made based on evaluations using an alternative metric on clustering performance, the Silhouette scores.”*

*The added supplementary section includes an explanation of the methodology, the Silhouette score calculations, and an interpretation of the results. We believe that i ncluding this metric enhances the robustness of our evaluation and provides a more comprehensive view of our clustering performance. Please refer to Additional File 1: Section S2.1 in the updated manuscript for a detailed discussion.*

3. While vmrseq provided superior performance in the nearest neighbor count score, what happens if no VMR selection is performed and a simple binning approach, such as in SINDBAD for example is used as input for dimensionality reduction, e.g. by PCA?

*Reply: Thank you for your valuable suggestion. First, we would like to emphasize that the primary objective of our proposed method is to identify genomic regions exhibiting i nter-cell heterogeneity with potential biological significance, rather than solely aiming for optimal clustering performance. Applying dimensionality reduction on genome-wide bins does not align with this primary goal. In particular, we would like to direct your attention to the following sentences in the Introduction section of our main text that state the main objective:*

*“To reflect these groupings of CpGs, identifying regions with distinctive methylation levels across cells, referred to as VMRs, is considered one of the main analytical objectives in the analysis of scBS-seq data. The identified variable regions may serve as epigenetic features of cell types and states and facilitate integrative analyses of single-cell multi-omics assays. They might also foster understanding of environmental influence, allowing the identification of epigenetic changes in response to extrinsic stimuli.”*

*We also added the following sentence to the main text to underline the emphasis:*

*“Although the primary objective of our proposed method is not to select features that optimize clustering performance, VMRs detected by vmrseq render reliable cell clusters.”*

*Further, in response to your suggestion, we have conducted a series of experiments to evaluate the efficacy of various region selection methods against a baseline approach similar to SINBAD* [*[ 2].*](https://www.zotero.org/google-docs/?BVD2rf) *Specifically, we employed simple imputation and principal component analysis (PCA) for dimensionality reduction on genome-wide 100 kb bins, following the methodology outlined in SINBAD. To facilitate a fair comparison that mitigates the curse of dimensionality, we also applied imputation and PCA to the detected regions by each method.*

*The results indicate that our region selection methods can maintain high clustering accuracy even with a greatly reduced feature set. This suggests that our methods effectively pinpoint biologically significant variability. We have added a new section in the supplementary materials (Additional File 1: Section S2.2) and a corresponding paragraph i n the main text discussing these experiments and their results. The added main text paragraph is as follows:*

*“We also conducted experiments contrasting the region selection methods to a baseline approach where no region selection was performed and principal component analysis (PCA) is used on genome-wide large-sized bins to perform dimensionality reduction, similarly as proposed in SINBAD. To ensure a fair comparison, PCA was also applied to the regions detected by each method. We observe that incorporating a dimension reduction step results in all methods achieving clustering performance comparable to that obtained using genome-wide information, despite the considerably smaller number of CpGs included in the detected regions (i.e., < 350K CpGs for vmrseq and > 1 million for all other methods). For a detailed discussion, please refer to Additional File 1: Section*

*S2.2.* ”

*We hope these additional analyses address your concern and further validate the robustness of our proposed methods.*

4. The selection of parameters, such as α = 0.05, minimum CpG counts = 5, …, is not described very well. The authors claim that the selection works well in practice, but no quantitative results are shown. A small simulation case showing selected evaluation metrics for a couple of different choices of parameters could strengthen that claim.

*Reply: We have addressed this concern by conducting a series of experiments to evaluate the impact of different parameter choices on our analysis. We used the experimental dataset from Luo et al* [*[3]*](https://www.zotero.org/google-docs/?1Zmon1) *i nstead of simulated data for benchmarking in the analysis to avoid possible bias from the pre-set simulation parameters. This sensitivity analysis examined the robustness of the nearest neighbor count score when varying the α and minimum CpG parameters. These results are now i ncluded in the supplementary materials (Additional File 1: Section S2.3). They demonstrated that α = 0.05 and minimum CpG counts = 5 are reasonable choices of hyperparameters, as these settings provided optimal clustering performance using minimal information. We also added the following sentence to the main text of our manuscript to direct readers to these supplementary results:*

*“Please refer to Additional File 1: Section S2.3 for a sensitivity analysis on these hyperparameters.”*

*We believe that this addition strengthens our claim and provides the quantitative evidence requested. We hope that these enhancements address your concerns and improve the clarity and rigor of our manuscript.*

5. vmrseq (stage 1 & 2) and the vmrseq CR (stage 1 only) algorithm provide very similar results in the simulation benchmarks (except for precision). This seems to be also true for real data (nearest neighbor score) and not only in the case of simulated chromosomes. However, the time complexity of optimizing a HMM and running it for each CR two times is probably much higher than stage 1 of the algorithm. Could stage 1 then not be enough for normal use-cases ?

*Reply: Thank you for your valuable suggestion. For users prioritizing speed over region ranking and high precision, we now recommend using the newly implemented option to disable stage 2 (i.e., applying only stage 1) in our package, which offers a more time-efficient computational approach compared to applying the full methodology. A corresponding revision has been made i n the manuscript, where we included the following sentences in the subsection ‘ Computational scalability of vmrseq’ :*

*“Therefore, when dealing with datasets consisting of a large number of cells, we recommend running only the first stage of the methodology for users who need a preliminary check or prioritize speed over high precision in region detection and the ranking of regions. This approach allows for a faster initial analysis before applying the full methodology.”*

**Minor:**

6. The variable C is introduced only in the time-complexity analysis but used already i n equation (1); Figure 1d is mentioned before Figure 1c.

*Reply: Thanks for your comments and we apologize for the errors. Annotation of the variable*

*has been added before equation (1), and figure numbering has been corrected.*

7. Several different variance cutoffs are used for multiple methods, but it is not explained why different cutoffs were used. Is that due to the difference in computing the variance? very different variance cutoffs

*Reply: Thanks for the comment and we apologize for the lack of clarity in our manuscript. Due to their distinct definitions, the ‘cutoffs’ of these methods are not comparable. Specifically, vmrseq uses variance. ‘Variance tαhreshold’ in scbs and smallwood represents the percentage of top-ranked a ‘variance quantile ’ that designates the upper quantile of an empirical null distribution of*

*windows in terms of high variance. scMET applies an upper limit on the expected false discovery rate to select highly variable windows. Therefore, in all our experiments except for the evaluation of the ratio of relevant areas under PR-curve (RRA), we used the default cutoffs recommended in the original publication of the methods. The range of thresholds used for RRA estimation was tailored to each method aiming to strike a balance between the computational time of the experiments and a PR-curve with steady and continuous progression. To further clarify the choices, we included the following* *sentences in the subsection ‘ Method i mplementations and parameter settings’ in our revised manuscript:*

*“We used default parameters as suggested in original publications of the evaluated methods in all our experiments. For RRA estimation, the sets of thresholds were tailored to each method to balance the trade-off between computational time and a PR-curve with steady and continuous progression.”*

8. The following section is discussed in the method section, but as it is an important l imitation and information for a user, this should be rather part of the discussion and not the methods: Only autosomes were included in this study since sex chromosomes have distinct methylation patterns (e.g., X-chromosome i nactivation) where vmrseq may not be applicable. Follow-up work is needed to examine whether model parameters for sex chromosomes shall differ from autosomes.

*Reply: Thank you for your comment. We agree and have moved the relevant paragraph from Methods section to Discussion to reflect its importance. Specifically, the paragraph is pasted as follows:*

*“Last but not least, users of vmrseq should note that our method has only been experimented with and evaluated on autosomes. Sex chromosomes might have distinct methylation patterns from autosomes due to cellular processes such as the X-inactivation. Follow-up work is needed to examine whether model parameters for sex chromosomes shall differ from autosomes.”*

9. Supplementary Figure 7-10 are very hard to read, due to the small font size.

*Reply: Thank you for your comment. We have updated Figure S7-S10 with a larger font size in our manuscript.*

10. Comment: The Repository vmrseq-experiments contains user-paths and deprecated code which probably should not be there.

*Reply: Thank you for your suggestion. We have cleared up the `vmrseq-experiments` Git repository by removing user paths and deprecated code scripts.*

# Reviewer #2

Reviewer #2: vmrseq is a novel tool that identifies candidate regions with variable methylation patterns across cells. The tool is an advancement over previous tools which require a predefined set of regions. Specifically, vmrseq uses an HMM to decode Unmethylated (U) and Methylated (M) regions. The authors compare vmrseq against other tools in both simulated and real datasets and show improved performance. The software was able to be ran on a toy dataset. Overall, the manuscript is well written, compelling, and will be of interest to the broader single cell community. I only had a few relatively minor questions.

*Reply: We thank the reviewer for providing supportive and constructive comments. We address all the questions and comments in our point-by-point response below.*

1. For the statement, "As a result, we remove the sites with an intermediate methylation level for every cell as a filtering step", does this mean that each item that is not 0 or 1 is considered missing?

*Reply: Yes. Specifically, prior to input to vmrseq, per cell we remove CpG sites with methylation l evel that is not 0 or 1. Hence they are considered as NA during model fitting. In datasets used in our case studies, the proportion of CpGs with intermediate methylation out of all sites with coverage in a cell is very small, ranging from 0 .33% to 0.55%. To further clarify, we included these numeric results in the Methods section of our manuscript by adding the following sentence:*

*“ As a result, we remove the sites with an intermediate methylation level from every cell as a filtering step,* *which affects a very small proportion of sites per cell (interquartile range*

*0.33%–0.55%) in the datasets used in our case studies*. ”

2. Although no formal p-value was derived for comparing the one-group vs. two group models, would it be possible to generate an empirical distribution via permutation of the CpG sites?

*Reply: Thank you for the suggestion. We had similar ideas during the development of the methodology and explored the possibility of deriving an empirical distribution via permutation of the CpG sites. However, a permutation of CpG sites across the genome would disrupt the inherent spatial correlation structure between CpG loci. The spatial correlation is a crucial aspect of bisulfite-sequencing data and must be maintained to produce a valid null distribution of the test statistic (i.e., the distribution when there is no VMR). The integrity of this spatial correlation is essential for accurately identifying variable methylation regions (VMRs). As a result, generating an empirical null distribution through permutation would not be straightforward or appropriate for our targeted test statistic, and thus, we did not pursue this approach.*

*To further clarify, we added the sentence to the Discussion section in the revised manuscript:*

*“We do not see a straightforward way to generate null distribution via either a theoretical or empirical approach.”*

3. Related to the results of Figure 3c,d, how were specific regions considered markers for a cell population? In standard single cell RNA-seq analysis, variable genes are chosen for clustering. However, a separate statistical test and metrices (e.g. Wilcoxon test in combination with a fold change cutoff) is used to find specific genes that are associated with each cluster. Can a similar paradigm be applied in the methylation clustering workflow?

*Reply: We apologize for not making this clear in the manuscript. We used cell type labels annotated by* [*[3]*](https://www.zotero.org/google-docs/?ixLgpe) *to determine marker regions. Specifically, marker regions of cell type mL4 among detected regions of each method are defined as those with absolute difference > 0.2 between average methylation levels of the targeted cell type and all other cell types. The above definition has been included in the caption of Figure 3d in the updated manuscript.*

*We deliberately avoided assessing statistical significance of VMR associations with cell types in order to avoid selection bias induced by ‘double-dipping’ - i.e. using the same data for cell type i dentification and inference of difference. This type of practice might result in inflation of Type I error rate and invalid inference* [*[4]*](https://www.zotero.org/google-docs/?AlOxqP)*.*

4. Related to Figure 4b, it was stated that "the VMRs seemed to contain some variation associated with cell cycle, where the cells in G2M phase displayed a more pronounced clustering tendency than the other two phases". While it does appear that there is some correlation with cell cycle, it does not seem like the primary driving factor as cells from different parts ~~are~~ (of) the cycle are represented i n each part of the UMAP. If clustering is performed on the RNA space, how does t he methylation clusters correlate with the RNA clusters?

*Reply: Yes, we agree that the cell cycle state does not appear to be a primary driving factor and we have revised the manuscript to clarify. Note that the cell cycle labels were estimated using the RNA-seq space only (see the subsection of Methods ‘ Multi-omics gastrulation dataset’ in our manuscript for details), thus we expect (and indeed confirmed) that there is variation of cell cycle in gene expression. The predicted cell cycle labels are used as reference to color the points in the UMAP plot of Figure 4b but the UMAP itself was constructed solely from DNAme i nformation. We are not aware of a straightforward way to correlate DNAme and RNA clusters from two different UMAPs. However, we explored the correspondences of methylation and gene expression more broadly and have made interesting discoveries (Figure 4c-e).*

5. The tool would be easier to install if it were deposited in a public R package repository such as CRAN or Bioconductor.

*Reply: Thank you for your valuable feedback. We are pleased to inform you that we have submitted the package to Bioconductor. Once the package is accepted and available on Bioconductor, users shall be able to install it conveniently using standard Bioconductor i nstallation commands.*

6. The online vignette is runnable using the supplied toy example. It would be nice to provide a vignette using a "real-world" dataset such as the ones analyzed in the paper which includes the downstream clustering analysis and annotation.

*Reply: Thank you for your valuable comment. Due to Bioconductor's file size restrictions (less than 5MB per package), it is not feasible to include large-scale real-world data in the vignette demonstration. To address this, we have created an additional GitHub repository that demonstrates the workflow for analyzing single-cell bisulfite sequencing data using a real-world example dataset. You can access this comprehensive example and the downstream clustering analysis and annotation at the following link:*

[*https://github.com/nshen7/vmrseq-workflow-vignette*](https://github.com/nshen7/vmrseq-workflow-vignette)*. We have also added a link to this repository on the front page of our package’s GitHub repository.*

# Reviewer #3

## Overall summary

The authors introduce vmrseq, a probabilistic approach to detect variable methylated regions (VMRs) associated cell-to-cell epigenetic heterogeneity. Unlike existing approaches, vmrseq does not rely on the use of a priori annotated regions (such as gene promoter) or sliding windows strategies. Instead, the authors i mplement a 2-stage approach in which an "scanning" step is first performed in order to identify candidate regions (CRs) in an unsupervised manner. In a second step, a hidden Markov model is used to flag VMRs among the pre-selected CRs (great to see that the authors discuss how the use of a 2-step approach prevents straightforward interpretation of p-values and error rates control). The performance of vmrseq was demonstrated using synthetic and real data, focusing on how the detection of VMRs can improve downstream clustering analyses.

The set of tools tailored to the properties of single cell methylation data vmrseq is somewhat limited. As such, vmrseq is a potentially important contribution in this area that complements existing approaches. However, as discussed below, some aspects of the methodology are unclear and the comparison with other approaches has some problematic aspects.

*Reply: We thank the reviewer for providing supportive and constructive comments. We address all the questions and comments in our point-by-point response below.*

## Major comments

1. Data pre-processing. a) Page 3 (lines 28-29) indicates that "sites with intermediate methylation level between 0 and 1 are filtered out for each cell". Does this mean that a different set of CpGs is considered for each cell? If so, how does this affect the construction of candidate regions? If the candidate regions are not consistent across cells, how does this affect the identification of VMRs? b) Page 14 (lines 2-3) mentions that missing values are allowed in the input matrix. How are missing values treated when constructing the candidate regions and performing VMR selection? c) The Methods states that the input for vmrseq is "processed and filtered whole-genome BSseq data". Beyond the filtering steps mentioned above, it would be helpful for potential users to have practical guidelines about more general quality control (e.g. to remove cells with poor data quality) that need to be applied prior to the use of vmrseq.

*Reply:* ***a)*** *We’d like to clarify that candidate regions are constructed based on the entire input dataset simultaneously and are consistent across cells. However, note that the sets of covered CpGs of each cell from sequencing are expected to be different due to the extremely low coverage of single-cell technologies. vmrseq evaluates the set of CpGs that are covered by a minimum number of cells across the input dataset (e.g., minimum n=5 by default and in our case studies). with numeric values ranging from zero to one, where 0 represents com×pletely unmethylated and The raw observations of methylation level are organized into a (# cell)	(# sites) data matrix*

*1 represents fully methylated. Sites that are not covered in a given cell are considered missing (i.e., filled by NA in the matrix). The filtering step in question essentially considers observations with an intermediate methylation value (i.e., greater than 0 and less than 1, indicating that not all reads covering that site in that cell had the same methylation status) in the data matrix as missing, and the removed sites usually take up a very small proportion out of all sites covered by a cell ( 0.33%–0.55% in our case studies ). Further, this filtering does not alter the set of CpGs that will be considered for inclusion in VMRs, except in the even more rare case that all cells covered at a particular CpG exhibited intermediate methylation (such CpGs would be removed entirely). To further clarify in the manuscript, we included the following sentence in the subsection ‘ Overview of vmrseq’ of Methods section:*

*“As a result, we remove the sites with an intermediate methylation level from every cell as a filtering step before input to vmrseq, which affects a very small proportion of sites per cell corresponding to the interquartile range of 0.33%–0.55% in the datasets used in our case studies.”*

b) *One of the strengths of vmrseq is that it naturally handles missingness in the input data matrix. Specifically, during CR construction, we only consider non-missing sites when computing the average methylation level across cells* [*(*](https://www.codecogs.com/eqnedit.php?latex=%5Cbar%7Bx%7D_%7Bk%5Ccdot%7D#0)*) , and compute the relative methylation levels (*

 *) for the covered sites in each cell. Then kernel smoothing and variance*

*estimation are applied to the computed relative methylation levels. During VMR identification, we directly model the per-site methylated cell count out of the total covered cell count in the sites) matrix. These details can be found in subsection ‘o verview of vmrseq’ in the Method×s input dataset - these* counts can be computed regardless of missing values in the (# cell)	( # section of our manuscript.

c) *In the experiments of our manuscript, we did not carry out these pre-processing steps but i nstead, adopted the processed .bam files provided by the original publications of the sequencing protocol and datasets* [*[3], [5], [6], [7]*](https://www.zotero.org/google-docs/?e5Kdtt) *(mentioned in the Methods section). However, by summarizing the best practice processing steps described in those publications as sources, we offer a flexible guideline intended to serve as a general reference rather than a strict requirement:*
   1. *Remove cells with a low non-conversion rate (e.g., ≤1% in mouse and ≤2% in human).*
   2. *. To eliminate contaminated samples, remove cells with a low number of non-clonal mapped reads (e.g., 400K in mouse; 500K in human).*
   3. *To protect against wells with multiple cells, remove cells exceeding an upper limit on coverage (≥ 15% of cytosines).*
   4. *R emove cells with low CpG site coverage (e.g., ≤ 50,000 CpG sites)*

*This paragraph has also been added to the vmrseq package online vignette*

*(* [*https://rpubs.com/nshen7/vmrseq-vignette )*](https://rpubs.com/nshen7/vmrseq-vignette) *as a practical guideline on quality control.*

2. Synthetic data benchmark. The broad conclusion was that the sliding window was the most critical hyper-parameter choice. In particular, approaches using Smallwood and scMET (larger window size) performed worse than scbs and vmrseq (small window size). This comparison is problematic as the performance of the methods is confounded by the choice of window/step size. If you were to use smaller regions as input for Smallwood and scMET (e.g. 2000-bp as in scbs), do they perform better? Similarly, if you use a larger window size for scbs, does it perform worse? This issue is accentuated by the simulation setting, where a large number of the simulated VMRs were shorted than the window size used by some approaches (Fig S2). To make the comparison more fair, I suggest to match the window/step size when comparing different approaches. You could e.g. present different results for a small, medium and a large sliding window size for all methods.

*Reply: Thank you for your insightful feedback. We intended to run all the methods with a smaller comparable window size, but unfortunately discovered that scMET was only able to be run on l arger window sizes. Specifically, we have tried to experiment with small window sizes such as 10kb, but the scMET software produced the following computational errors:*

*Error in sampler$call_sampler(c(args, dotlist)) :*

*Exception: Error transforming variable w_gamma: lub_free: Bounded variable is -11.2741, but must be in the interval [-10, 10] (in 'model_scmet' at line 86)*

*We are unaware of the exact reason behind the errors, but we speculate that this is due to i nappropriate model specifications for a smaller window size like 10kb. Because of this sensitivity of performance of some methods with window size, for a fair comparison across methods, we opted for window sizes that are recommended by the original publication of the methods* [*[ 8], [9], [10] .*](https://www.zotero.org/google-docs/?opgYXc) *The choice of 2000-bp of vmrseq is justified by the correlation pattern between CpGs, as mentioned in the manuscript. Specifically, a window size of 2,000 base pairs (bp) was used in CR construction because the average correlation between CpG sites was observed to slowly decay as CpG-CpG distance grows and reaches a plateau at around 2,000 bp. This has been demonstrated in Additional File 1: Figure S17.*

3. Analysis of the mouse frontal cortex dataset. a) The issues highlighted in the comment above also apply here. Again, the performance of the approaches shown in Figure 3b correlates with the choice of sliding window size. A fairer comparison will account for this by comparing performance of each method after matching window size. b) Note that Kapourani et al analysed the same data using scMET and found better performance when using a more targeted approach in which input features were defined by a set of pre-annotated regions (e.g. gene promoters rather than a window size approach). It may be worth discussing such results and how a targeted approach (e.g. looking for VMRs only within gene promoters) could potentially improve performance across all methods (e.g. in Kapourani et al, methylation patterns within distal H3K27ac and H3K4me1 peaks better characterised the sub-population structure in these data when comparing against gene promoters). c) Similarly, the screening step used by vmrseq to

i dentify CRs could be used as a pre-filtering step for other approaches too avoiding the use of sliding window approaches (of course, as the authors note, this would affect the interpretation of p-values, etc). It would be helpful to understand what step is more important: the initial screening step to identify CRs or the 2nd step (i.e. the HMM in vmrseq)?

*Reply:*

a) *As mentioned in our response to question #2, the choice of window size is limited for scMET due to the fact that their software does not accommodate small window sizes. Thus we applied the recommended parameters as indicated in the methods’ original publication to ensure a consistent experimental methodology.*
b) *Thank you for your valuable suggestion. vmrseq is not applicable on targeted regions such as histone peaks due to their small sizes preventing the kernel smoothing step in stage 1. Therefore, we conducted an analysis to evaluate the clustering performance of variable methylation regions (VMRs) overlapping with these specific features, including histone modifications and gene promoters. In this analysis, we found that distal H3K27ac and H3K4me1 peaks outperformed gene promoters in terms of clustering performance for cell subtypes, which aligns with the findings in scMET* [*[8]*](https://www.zotero.org/google-docs/?7nkTNy) *. However, none of the targeted regions achieved a score as high as that obtained by using all VMRs, highlighting the value of our genome-wide approach. We have made revisions in the main text to include the results of this analysis. Specifically, we added the following sentences:*

*“In addition to genome-wide VMRs, we evaluated the clustering performance of VMRs that overlap with specific features such as histone modifications and gene promoters (Additional File 1: Table S1). All feature types achieved a perfect nearest neighbor count score for broad class l abels. For the subtypes, distal H3K27ac and H3K4me1 peaks demonstrated superior clustering performance compared to gene promoters for the cell subtypes, however, none of the targeted regions achieved a score as high as that obtained by using all VMRs.”*

c) *Yes, since the candidate regions are a selected set of CpG sites that are biased toward higher variation in methylation compared to the rest CpG sites in the genome, applying hypothesis testing on them might be misleading even with multiple testing corrections. In particular, it could result in an increased likelihood of false positives and reduced power to detect true positives. Regarding the relative importance of the two stages in vmrseq, we believe they have distinct objectives, yet are functionally coupled and achieve optimal performance when employed together. In particular, stage 1 narrows down the search for VMRs from the entire genome to a set of candidate regions, which usually has a high recall of true positives. Then stage 2 further refines the set of selected regions to elevate the precision (as demonstrated in our simulation studies) and provide a ranking of VMRs statistically robust to the sparsity and spatial correlation between CpGs.*

*For users prioritizing speed over region ranking and high precision, we also provide the option to disable stage 2 (i.e., applying only stage 1) in our package, which offers a more time-efficient computational approach compared to applying the full methodology. A corresponding revision has been made in the manuscript, where we included the following sentences in the subsection ‘ Computational scalability of vmrseq’ :*

*“Therefore, when dealing with datasets consisting of a large number of cells, we recommend running only the first stage of the methodology for users who need a preliminary check or prioritize speed over high precision in region detection and the ranking of regions. This approach allows for a faster initial analysis before applying the full methodology.”*

4. Use of an annotated dataset to infer vmrseq parameters: a) As noted in the discussion, the need of pre-annotated data can be problematic. As the same dataset was used for hyper-parameter tuning and benchmark, could this also affect the comparison shown in Figure 3b? Perhaps you could perform a sensitivity analysis to see how the results change if a different dataset the Luo et al data is excluded for hyper-parameter tuning (i.e. you only use the Liu et al data)? b) Currently, all the information regarding the annotated dataset is provided in the supplement. It would be helpful to have a brief description in the main text. c) When training the relevant models, ~~who~~ (how) were the Luo et al and Lio et al datasets combined? Simple merge of all cells from both datasets?

*Reply:*

a) *Thank you for the suggestion. We conducted a sensitivity analysis by examining the difference between the sets of estimated parameters before and after excluding the Luo et al.* [*[ 3]*](https://www.zotero.org/google-docs/?IC0coz) *dataset from training. In the analysis, we demonstrated that the clustering performance of VMRs i s robust to hyperparameters when cells are labeled by broad classes but varies with subtype l abels; and an alpha value of 0.05 and a minimum CpG count of 5 are reasonable choices based on the results. Please refer to section S1.1.4 in Additional File 1 of the updated manuscript for a detailed discussion of the results.*
b) *Thank you for the suggestion. We have added the subsection ‘ Annotated datasets used for empirical parameter estimation’ in the Methods section of the main text, which provides a brief description of the Luo et al* [*[ 3]*](https://www.zotero.org/google-docs/?1djcUn) *and Liu et al* [*[6]*](https://www.zotero.org/google-docs/?cxDCcv) *datasets used for training the model parameters.*
c) *Prior to model training, within each dataset, we first aggregated the cells in each annotated subtype into a pseudo-bulk sample (as described in Additional File 1: Section S1.1). Luo et al* [*[ 3]*](https://www.zotero.org/google-docs/?PNOMqo) *and Liu et al* [*[6]*](https://www.zotero.org/google-docs/?jl3cy1) *applied different methodologies to annotate the cell subtypes and rendered completely non-overlapping names of the subtypes. Therefore, we consider all pseudo-bulk samples from subtypes of these two datasets as independent and equally weighted samples.*

*To estimate parameters in the transition probability distribution, we included in the training data*

*CpG pairs from each pseudo-bulk sample that are not farther apart than 10 sites, where one CpG pair of one pseudo-bulk sample is considered as one observation. To estimate parameters i n the emission probability distribution, we subsampled 10,000 CpG sites from each pseudo-bulk sample and trained the ZIBB/BB models, where one CpG site of one pseudo-bulk sample is considered as one observation.*

5. Some aspects of the method are unclear and a substantial part of the methodological details are only provided in the supplement. This makes the paper harder to read. a) Identification of candidate regions: when defining "5 adjacent CpGs", is there a minimum distance between the CpGs to be considered within the same CR? b) Estimation of parameters for the "Empirical probability" model. It would be helpful to explicitly mention what parameters are estimated using the annotated data and a brief description of how these were obtained: maximum l ikelihood? c) Page 16. Whilst it is easy to understand what do the hidden states represent in the one-grouping model specification, this is less clear for the two-grouping model specification. Please clarify the definition of bi-variate hidden states: what does each component of the vector $s_k$ represent? d) Page 18. VMRs are defined for regions with a minimum 5 sites exhibiting the hidden state (0,1). The authors state that the number of sites (5 in their case) can be customised depending on the biological question. Please provide some guidance (examples?) of how this would be done. e) Equation (5) is the ZIBB model trained i n the same way as for the one-grouping model?

*Reply: We apologize for the lack of clarity in our manuscript. We respond to each query and comment i n our detailed point-by-point reply below.*

a) *No, there is no minimum distance between the CpGs to be considered within the same CR. However, we set the maximum distance between the CpGs in the same CR to be 2,000 bp because the correlation between CpG sites does not significantly increase when the distance between CpG-CpG pairs exceeds 2,000 base pairs (Additional File 1: Figure S17). To improve clarity, we have included the following sentence in the Methods section of our manuscript:*

*“The maximum distance between two adjacent CpG sites is set to 2,000 bp for the same reason of determining the default smoothing bandwidth.”*

b) *Thank you for the suggestion. We have made revisions accordingly in sections S1.1.2-S1.1.3 i n the supplementary materials of our manuscript to clarify the set of estimated parameters and the method used for estimation. In summary, the transition probabilities* [*(*](https://www.codecogs.com/eqnedit.php?latex=P_d(s_k%20%3D%200%7Cs_%7Bk-1%7D%20%3D%200)#0) *,*

*,*	[*)*](https://www.codecogs.com/eqnedit.php?latex=P_d(s_k%20%3D%201%7Cs_%7Bk-1%7D%20%3D%201)#0) *with respect to a*

a

n

d

*between-CpG distance* [*(*](https://www.codecogs.com/eqnedit.php?latex=d%20%3D%202%2C%203%2C%204%2C%20...%2C%202000#0) *bp) were estimated by LOESS using local fitting.*

*Regression intercepts and cubic spline function*

[,](https://www.codecogs.com/eqnedit.php?latex=%5C%7B%5Cbeta%5EU_%7B%5Cnu%2C%200%7D%2C%20%5Cbeta%5EU_%7B%5Cmu%2C%200%7D%2C%20%5Cbeta%5EU_%7B%5Csigma%2C%200%7D%5C%7D#0)

s

l

o

p

e

s

*i n the ZIBB regression were estimated using maximum likelihood. Similarly, intercepts in*

*and* *i n the intercept-only BB regression were estimated via maximum likelihood. Please refer to the red texts in our updated supplementary materials for details of the changes.*

c) *Thank you for the suggestion. We have made revisions in the subsection ‘ Two-grouping model specification’ to clarify the definition of bi-variate hidden states. In short, recalling the assumption of U and M grouping (Fig. 1d in main text), the* *i n the bi-variate hidden state of*

*site* *represents the underlying state of the U grouping, and* *the underlying state of the M grouping. Please refer to the red texts in our updated manuscript for more details.*

d) *Thank you for the suggestion. We have made revisions in the Methods section to include examples where alternative thresholds on minimum number of CpGs in a VMR can be used. Specifically, we added the following sentence:*

*“For example, a higher threshold (e.g., ≥ 50 CpGs) for capturing broad epigenetic changes in cancer studies* [*[ 11] ,*](https://www.zotero.org/google-docs/?eiCwEe) *and a lower threshold (e.g., 5-10 CpGs) for detecting fine-scale regulatory changes in developmental studies* [*[12]*](https://www.zotero.org/google-docs/?A895yk)*. ”*

e) *Yes, the one-grouping and two-grouping models share the model structure and trained parameters. To improve clarity, we added the following sentence in the subsection ‘ Two-grouping model specification’ of our manuscript's main text:*

*“The BB and ZIBB models share the same structure and estimated parameters as in the one-grouping case.”*

6. It would be helpful for users to understand the computational requirements. How do the different approaches compare in terms of scalability?

*Reply: Thank you for your suggestion. We have addressed this concern by conducting a detailed scalability analysis using synthetic data under various settings. The results of this analysis are now* *included in the main text of our manuscript (subsection “ Computational scalability of vmrseq ”). Specifically, the subsection is as follows:*

*“We conducted a scalability analysis using synthetic data to evaluate the running times of various methods across different settings (Additional File 1: Fig. S19). Due to the second-order computational complexity with respect to the number of cells, the running time of vmrseq exhibits a quadratic increase as cell count grows, while vmrseq CRs, scMET, and scbs present a linear increase. Therefore, when dealing with datasets consisting of a large number of cells, we recommend running only the first stage of the methodology for users who need a preliminary check or prioritize speed over high precision in region detection and the ranking of regions. This approach allows for a faster initial analysis before applying the full methodology.”*

*We believe that this addition provides the necessary quantitative evidence to help users understand the computational requirements and scalability of the different approaches. This i nformation will assist users in making informed decisions based on their computational resources and analysis needs. We hope that this enhancement addresses your concern and i mproves the overall clarity and utility of our manuscript.*

## Minor comments

7. I mprovements for some Figures: a) Figure 1b: define colour-scale for yellow l ollipop. Missing? b) Caption of Fig 1d. Unclear how the "homogenous cell population" was defined here. Is this synthetic data? Real data subsetted in some way? c) Figure S7 is too small and difficult to read without zooming in.

*Reply:*

a) *Thank you for your suggestion. In Figure 1b, the definition for the color yellow was originally at the bottom left of the rectangle of the first candidate region in Stage 1, which i ndeed can be easily overlooked. We have moved the annotation to the top left of the rectangle to enhance its visibility.*
b) *We apologize for the confusion caused. The homogeneous cell population was subsetted from real data, specifically, the Luo et al data* [*[3].*](https://www.zotero.org/google-docs/?YFpJKS) *To improve clarity, we have i ncluded the following sentence to the caption of Figure 1c in our updated manuscript:*

*“Cells from the subtype mL4 (n=370) in the annotated single-cell bisulfite sequencing dataset published by Luo et al. were selected to establish a homogeneous cell population, which served as the input for the analytical methods.”*

c) *Thank you for your suggestion. We have updated Figure S7-S10 with a larger font size i n our manuscript.*

8. I was able to install the R library from GitHub with no problem. I was also able to run the vignette. However, I got a couple of warnings after running results <- vmrseq.fit(gr) . Not sure this affects the performance of vmrseq, but worth checking why do they appear. *Warning messages: 1: I n split.default(which(direction > 0), segments[direction > 0]) : data length is not a multiple of split variable*

*Reply: Thank you for your valuable feedback and for pointing out the warning messages encountered while running the vmrseq.fit(gr) function. This warning occurred due to the absence of a filtering step that remove sites from the toy dataset with low cell coverage, which caused NA(s) i n the smoothed variance values. We have since included the filtering step in the package vignette. The performance of vmrseq in our manuscript was not affected since the filtering step was performed prior to all experiments in the studies. Further, we have enhanced our package i mplementation to accommodate the presence of NA values of variance, and have confirmed that the warnings no longer appear even without the filtering step.*

[1] [*P. J. Rousseeuw, “Silhouettes: A graphical aid to the interpretation and validation of cluster analysis,” J. Comput. Appl. Math. , vol. 20, pp. 53–65, 1987, doi:*](https://www.zotero.org/google-docs/?SgfrRe)

[*https://doi.org/10.1016/0377-0427(87)90125-7.*](https://www.zotero.org/google-docs/?SgfrRe)

[2] [*Y. Uzun, W. Yu, C. Chen, and K. Tan, “SINBAD: a flexible tool for single cell DNA methylation data.” bioRxiv, p. 2021.10.23.465577, Oct. 24, 2021. doi:*](https://www.zotero.org/google-docs/?SgfrRe)

[*10.1101/2021.10.23.465577.*](https://www.zotero.org/google-docs/?SgfrRe)

[3] [*C. Luo et al. , “Single-cell methylomes identify neuronal subtypes and regulatory elements in mammalian cortex,” Science , vol. 357, no. 6351, pp. 600–604, Aug. 2017, doi:*](https://www.zotero.org/google-docs/?SgfrRe)

[*10.1126/science.aan3351.*](https://www.zotero.org/google-docs/?SgfrRe)

[4] [*L. L. Gao, J. Bien, and D. Witten, “Selective Inference for Hierarchical Clustering,” J. Am.*](https://www.zotero.org/google-docs/?SgfrRe)

[*Stat. Assoc. , vol. 119, no. 545, pp. 332–342, Jan. 2024, doi:*](https://www.zotero.org/google-docs/?SgfrRe)

[*10.1080/01621459.2022.2116331.*](https://www.zotero.org/google-docs/?SgfrRe)

[5] [*C. Luo et al. , “Robust single-cell DNA methylome profiling with snmC-seq2,” Nat. Commun. , vol. 9, no. 1, p. 3824, Sep. 2018, doi: 10.1038/s41467-018-06355-2.*](https://www.zotero.org/google-docs/?SgfrRe)
[6] [*H. Liu et al. , “DNA methylation atlas of the mouse brain at single-cell resolution,” Nature, vol. 598, no. 7879, Art. no. 7879, Oct. 2021, doi: 10.1038/s41586-020-03182-8.*](https://www.zotero.org/google-docs/?SgfrRe)
[7] [*R. Argelaguet et al. , “Multi-omics profiling of mouse gastrulation at single-cell resolution,” Nature , vol. 576, no. 7787, Art. no. 7787, Dec. 2019, doi: 10.1038/s41586-019-1825-8.*](https://www.zotero.org/google-docs/?SgfrRe)
[8] [*C.-A. Kapourani, R. Argelaguet, G. Sanguinetti, and C. A. Vallejos, “scMET: Bayesian modeling of DNA methylation heterogeneity at single-cell resolution,” Genome Biol. , vol. 22, no. 1, p. 114, Apr. 2021, doi: 10.1186/s13059-021-02329-8.*](https://www.zotero.org/google-docs/?SgfrRe)
[9] [*L. P. M. Kremer, L. Küchenhoff, S. Cerrizuela, A. Martin-Villalba, and S. Anders, “Analyzing single-cell bisulfite sequencing data with scbs (old version of MethSCAn).” bioRxiv, p.*](https://www.zotero.org/google-docs/?SgfrRe)

[*2022.06.15.496318, Jun. 17, 2022. doi: 10.1101/2022.06.15.496318.*](https://www.zotero.org/google-docs/?SgfrRe)

[10] [*S. A. Smallwood et al. , “Single-cell genome-wide bisulfite sequencing for assessing epigenetic heterogeneity,” Nat. Methods , vol. 11, no. 8, Art. no. 8, Aug. 2014, doi:*](https://www.zotero.org/google-docs/?SgfrRe)

[*10.1038/nmeth.3035.*](https://www.zotero.org/google-docs/?SgfrRe)

[11] [*W. Timp et al. , “Large hypomethylated blocks as a universal defining epigenetic alteration in human solid tumors,” Genome Med. , vol. 6, no. 8, p. 61, Aug. 2014, doi:*](https://www.zotero.org/google-docs/?SgfrRe)

[*10.1186/s13073-014-0061-y.*](https://www.zotero.org/google-docs/?SgfrRe)

[12] [*W. Chang et al. , “Dynamic changes in whole genome DNA methylation, chromatin and gene expression during mouse lens differentiation,” Epigenetics Chromatin , vol. 16, no. 1, p. 4, Jan. 2023, doi: 10.1186/s13072-023-00478-7.*](https://www.zotero.org/google-docs/?SgfrRe)

**Second round of review**

**Reviewer 1**

2. Synthetic data benchmark. The broad conclusion was that the sliding window was the most critical hyper-parameter choice. In particular, approaches using Smallwood and scMET (larger window size) performed worse than scbs and vmrseq (small window size). This comparison is problematic as the performance of the methods is confounded by the choice of window/step size. If you were to use smaller regions as input for Smallwood and scMET (e.g. 2000-bp as in scbs), do they perform better?
Similarly, if you use a larger window size for scbs, does it perform worse? This issue is accentuated by the simulation setting, where a large number of the simulated VMRs were shorted than the window size used by some approaches (Fig S2). To make the comparison more fair, I suggest to match the window/step size when comparing different approaches. You could e.g. present different results for a small, medium and a large sliding window size for all methods.

*Response: Thank you for your insightful feedback. We intended to run all the methods with a smaller comparable window size, but unfortunately discovered that scMET was only able to be run on larger window sizes. Specifically, we have tried to experiment with small window sizes such as 10kb, but the scMET software produced the following computational errors:
Error in sampler$call_sampler(c(args, dotlist)) :
Exception: Error transforming variable w_gamma: lub_free: Bounded variable is -11.2741, but must be in the interval [-10, 10] (in 'model_scmet' at line 86)*
*We are unaware of the exact reason behind the errors, but we speculate that this is due to inappropriate model specifications for a smaller window size like 10kb. Because of this sensitivity of performance of some methods with window size, for a fair comparison across methods, we opted for window sizes that are recommended by the original publication of the methods [8], [9], [10]. The choice of 2000-bp of vmrseq is justified by the correlation pattern between CpGs, as mentioned in the manuscript.
Specifically, a window size of 2,000 base pairs (bp) was used in CR construction because the average correlation between CpG sites was observed to slowly decay as CpG-CpG distance grows and reaches a plateau at around 2,000 bp. This has been demonstrated in Additional File 1: Figure S17.*

Response to response: I really don’t think this is an adequate answer to the point raised. Even if scMET doesn’t allow for smaller windows, other tools do. A proper comparison would be with identical or at least similar window sizes and I don’t really understand why the authors have not complied with this request.

**Reviewer 2**

The authors have addressed my concerns and improved the clarity of the manuscript. The submission of the package to Bioconductor will greatly improve the usage of the tool.

**Reviewer 3**

The authors have thoroughly revised the manuscript and addressed my comments. At this stage, my only remaining comment relates to the answer provided to major comment 2 from my original report. The discussion provided about the importance of the sliding window size is important and that something along those lines should be added to the Discussion section. Even if some of the existing approaches do not allow users to specify smaller window sizes, as it can help users to better understand the findings generated by each method and can inform future methodology development.

**Authors Response**

**Point-by-point responses to the reviewers’ comments:**

Reviewer #1:

Regarding 2nd major comment: I really don’t think this is an adequate answer to the point raised. Even if scMET doesn’t allow for smaller windows, other tools do. A proper comparison would be with identical or at least similar window sizes and I don’t really understand why the authors have not complied with this request.

*Response:
Thank you for the valuable suggestion. We have conducted an additional set of experiments on both synthetic datasets and the mouse frontal cortex dataset to directly compare the performance of Smallwood with 2-kb windows (new) and 3-kb windows (default). We observed that a all results were very similar for these two window sizes, and that the smaller window size only marginally improves the performance of the method (see Additional File 1: Figures S24-28). Moreover, Smallwood still did not achieve any comparable level of accuracy and clustering performance to vmrseq and scbs when using the same window size. A new section has been added in the supplementary materials (see Additional File 1: Section S2.1) to discuss these additional experiments in detail. We also revised the paragraph in the main text regarding the discussion about window size and bandwidth to the following to briefly summarize the results of the additional experiments (colored red in the manuscript):*
 *It may appear that a larger size of sliding windows (i.e. 3kb used by Smallwood and 20kb used by scMET) results in reduced performance compared to a smaller smoothing bandwidth or window size (i.e. 2kb bandwidth used by vmrseq and 2kb windows used by scbs). However, we conducted an additional set of experiments on both synthetic datasets and the mouse frontal cortex dataset showing that a smaller window size alone does not seem to meaningfully improve the accuracy of detecting VMRs (see Additional File 1: Figures 24-28 and Section S2.1 for a detailed discussion).*

*We also included the following sentence in the subsection of ‘Method implementations and parameter settings’ in the “Methods” section of the manuscript for clarification:

However, we performed additional experiments using the same 2,000-bp window size as vmrseq and scbs to assess the impact of varying window sizes on method performance (see Additional File 1: Section S2.1 for a detailed discussion).
We hope these additional analyses address your concern and further validate the robustness of our proposed methods.*

Reviewer #2:

The authors have addressed my concerns and improved the clarity of the manuscript. The submission of the package to Bioconductor will greatly improve the usage of the tool.

*Response:
Thank you for your thoughtful review. We greatly appreciate the time and effort you have invested in evaluating our work.*

Reviewer #3:

The authors have thoroughly revised the manuscript and addressed my comments. At this stage, my only remaining comment relates to the answer provided to major comment 2 from my original report. The discussion provided about the importance of the sliding window size is important and that something along those lines should be added to the Discussion section. Even if some of the existing approaches do not allow users to specify smaller window sizes, as it can help users to better understand the findings generated by each method and can inform future methodology development.

*Response:
Thank you for the valuable advice. On account of a suggestion from another reviewer, we further explored the impact of changing the window size of Smallwood from the default 3 kb to 2 kb, which matches the bandwidth used for vmrseq and scbs. Consequently, we found that a smaller window size only slightly enhances the method's effectiveness and does not reach a comparable level of accuracy or clustering performance when compared to vmrseq and scbs. This suggests that window size/bandwidth alone does not explain the observed performance differences and does not support our previous hypothesis that the performance is largely related to window size/bandwidth. To update the results in our manuscript, a new section has been added to the supplementary materials (see Additional File 1: Section S2.1) presenting these additional experiments in detail. We also revised the paragraph in the main text regarding the discussion about window size and bandwidth to the following (colored red in the manuscript):*

*It may appear that a larger size of sliding windows (i.e. 3kb used by Smallwood and 20kb used by scMET) results in reduced performance compared to a smaller smoothng bandwidth or window size (i.e. 2kb bandwidth used by vmrseq and 2kb windows used by scbs). However, we conducted an additional set of experiments on both synthetic datasets and the mouse frontal cortex dataset showing that a smaller window size alone does not seem to meaningfully improve the accuracy of detecting VMRs (see Additional File 1:* *Figures 24-28 and Section S2.1 for a detailed discussion).

We also included the following sentence in the subsection of ‘Method implementations and parameter settings’ in the “Methods” section of the manuscript:

However, we performed additional experiments using the same 2,000-bp window size as vmrseq and scbs to assess the impact of varying window size on method performance (see Additional File 1: Section S2.1 for a detailed discussion).
We hope these additional analyses address your concern.*

**Third round of review**

**Reviewer 1**

This manuscript by Shen & Korthauer introduces vmrseq, a statistical method for detecting variably methylated regions (VMRs) in single-cell DNA methylation data. The authors present a two-stage approach that overcomes limitations of previous methods by identifying VMRs at base-pair resolution without relying on predefined genomic windows or sliding windows of fixed sizes. This leads to reduction of false positive CpG sites within VMRs as demonstrated in the manuscript. The authors benchmarked vmrseq on simulated and real datasets. vmrseq outperforms existing methods in accuracy of VMR detection on simulated data and improves downstream analyses such as cell type clustering on real data. Overall, vmrseq addresses an important step in the analysis of single-cell DNA methylation.

This manuscript is in revision and I have gone through the last available review reports and the authors’ responses. The authors have made substantial revisions to address previous reviewer comments, added new experiments and discussion testing the impact of base-pair window sizes on performance. I have no additional comments.

**Reviewer 3**

No further comments.
